# Oprozomib in patients with newly diagnosed multiple myeloma

**DOI:** 10.1038/s41408-019-0232-6

**Published:** 2019-08-16

**Authors:** Parameswaran Hari, Jeffrey V. Matous, Peter M. Voorhees, Kenneth H. Shain, Mihaela Obreja, John Frye, Hisaki Fujii, Andrzej J. Jakubowiak, Davide Rossi, Pieter Sonneveld

**Affiliations:** 1 Medical College of Wisconsin, Milwaukee, WI USA; 2 Colorado Blood Cancer Institute, Denver, CO USA; 3 Levine Cancer Institute, Atrium Health, Charlotte, NC USA; 4 Moffitt Cancer Center, Tampa, FL USA; 5 Amgen Inc., Thousand Oaks, CA USA; 6 University of Chicago Medical Center, Chicago, IL USA; 7 University of Eastern Piedmont, Novara, Italy; 8 Erasmus MC Cancer Institute, Rotterdam, Netherlands

**Keywords:** Cancer, Medical research

Dear Editor,

Treatment options for newly diagnosed multiple myeloma (NDMM) commonly include triplet regimens using the first-in-class proteasome inhibitor (PI) bortezomib, a corticosteroid, and an alkylator or immunomodulator^1,2^. Patients ultimately relapse with these regimens, and bortezomib is associated with peripheral neuropathy (PN)^3^. Additional effective, well-tolerated, and convenient frontline treatment options are needed.

Carfilzomib is an intravenously administered, second-generation, irreversible tetrapeptide PI approved for relapsed or refractory multiple myeloma (RRMM)^4^. In frontline studies, carfilzomib treatment resulted in hiqh-quality responses with low PN rates^5–8^, demonstrating therapeutic potential of second-generation, irreversible PIs in this setting.

Oprozomib, a tripeptide analog of carfilzomib, is an orally bioavailable PI^9^. Oprozomib has shown antitumor activity comparable with carfilzomib in preclinical models^9^. In RRMM patients, oprozomib demonstrated promising efficacy when used as a single-agent or in combination therapy^10–12^. As continuous/extended therapy has become increasingly important in NDMM, there is a need for treatments with convenient dosing, such as all-oral regimens. Oprozomib is expected to be a convenient treatment option, especially in all-oral regimens.

We conducted two phase 1b/2 studies evaluating three oprozomib-based regimens for NDMM: the OPZ003 study evaluated oprozomib-dexamethasone with lenalidomide or cyclophosphamide (ORd or OCyd, respectively); the OPZ006 study evaluated oprozomib-melphalan-prednisone (OMP).

OPZ003 (NCT01881789) and OPZ006 (NCT02072863) were multicenter, open-label, phase 1b/2 studies. Adult, transplant-ineligible NDMM patients were eligible. The primary objective of the phase 1b portions was to determine oprozomib’s maximum tolerated dose (MTD) using a standard 3 + 3 dose escalation schema. Efficacy was a primary objective of the phase 2 portions. Disease response was assessed by investigators according to International Myeloma Working Group – Uniform Response Criteria. Duration of response (DOR) was defined as time from achievement of a partial response (PR) or better to confirmed disease progression or death due to any cause. Progression-free survival (PFS) was defined as time from treatment initiation to disease progression or death due to any cause. Both protocols were approved by institutional review boards and patients provided written informed consent.

In OPZ003, ORd patients received oprozomib orally on days 1 through 5 and days 15 through 19 (5/14 schedule) or on days 1, 2, 8, 9, 15, 16, 22, and 23 (2/7 schedule) of every 28-day cycle. OCyd patients received the 2/7 schedule only (Fig. S1). The starting oprozomib dose in OPZ003 was 210 mg. ORd patients received oral lenalidomide 25 mg on days 1–21. OCyd patients received oral cyclophosphamide 300 mg/m^2^ on days 1, 8, and 15. Dexamethasone 20 mg was given on the 2/7 schedule. Treatment was administered until disease progression, unacceptable toxicity, or for 24 (ORd) or 8 (OCyd) cycles, whichever occurred first. Concomitant medications (including anti-diarrheals/anti-emetics) are described in the supplement.

In OPZ006, patients received oprozomib orally on days 1–5, days 15–19, and days 29–33 with melphalan 9 mg/m^2^ and prednisone 60 mg/m^2^ on days 1–4 of a 42-day cycle. The starting oprozomib dose was 180 mg. OMP was administered until disease progression, unacceptable toxicity, or nine cycles, whichever occurred first.

Twenty-two patients were enrolled in OPZ003, and 21 treated. Median age was 67 years (range, 54–79) (Table S1). At data cut-off, seven OPZ006 patients were treated. Median age was 71 years (range 66–84). Eighteen OPZ003 patients and five OPZ006 patients discontinued treatment (reasons given in Table S2).

The ORd arm of the OPZ003 study started with the 5/14 schedule, and the first dosing level tested was 210 mg (*n* = 3). This schedule required two preplanned dose de-escalations to 180 mg and then subsequently to 150 mg due to DLTs (Table S3). Because of these results with the 5/14 schedule, the protocol was amended and the ORd regimen enrolled two cohorts with the 2/7 schedule: a starting cohort at 210 mg and an escalation to 240 mg where one DLT occurred (Table S3). Comparatively, the OCyd arm started with the 2/7 schedule with a dose of 210 mg. A program evaluation identified that the safety profile and pharmacokinetic (PK) characteristics of the formulation used in all oprozomib studies required further optimization and thus enrollment in OPZ003 was halted during dose-escalation. The MTD of oprozomib for ORd or OCyd could not be established because there were not enough data for MTD determination. Only OPZ006 patients enrolled in the first dosing cohort (180 mg; *n* = 7) were treated before the study was terminated based on sponsor decision to not pursue the OMP combination; thus the MTD of oprozomib for OMP could also not be established.

In OPZ003, mean duration of oprozomib administration was 16.2 weeks (range, 0.6‒97.7) on the 5/14 schedule in the ORd arm, 36.6 weeks (range, 0.3‒72.3) on the 2/7 schedule in the ORd arm, and 42.0 weeks (range, 15.3‒64.3) on the 2/7 schedule in the OCyd arm. All patients reported a treatment-emergent adverse event (TEAE; Table 1). Grade ≥ 3 TEAEs were reported for 12 patients (92.3%) on the 5/14 schedule in the ORd arm, five patients (100%) on the 2/7 schedule in the ORd arm, and two patients (66.7%) on the 2/7 schedule in the OCyd arm. AE-related discontinuations occurred in four patients (30.8%) on the 5/14 schedule in the ORd arm and two patients (40.0%) on the 2/7 schedule in the ORd arm (supplement). No patient on the 2/7 schedule in the OCyd arm discontinued due to AEs.

**Table 1:** Summary of the most common treatment-emergent adverse events

	ORd				OCyd		OMP	
*n* (%)	5/14 Schedule, 150–210 mg OPZ *N* = 13		2/7 Schedule, 210 or 240 mg OPZ *N* = 5		2/7 Schedule, 210 mg OPZ *N* = 3		2/7 Schedule, 180 mg OPZ *N* = 7	
	Any grade	Grade 3 or greater	Any grade	Grade 3 or greater	Any grade	Grade 3 or greater	Any grade	Grade 3 or greater
Nausea	11 (84.6)	0	5 (100.0)	0	2 (66.7)	0	5 (71.4)	1 (14.3)
Cough	2 (15.4)	0	5 (100.0)	0	1 (33.3)	0	0	0
Diarrhea	11 (84.6)	2 (15.4)	4 (80.0)	0	2 (66.7)	0	6 (85.7)	1 (14.3)
Fatigue	7 (53.8)	1 (7.7)	4 (80.0)	1 (20.0)	1 (33.3)	1 (33.3)	2 (28.6)	0
Vomiting	7 (53.8)	0	4 (80.0)	0	0	0	4 (57.1)	1 (14.3)
Dizziness	7 (53.8)	1 (7.7)	3 (60.0)	0	1 (33.3)	0	1 (14.3)	1 (14.3)
Constipation	6 (46.2)	0	3 (60.0)	0	0	0	3 (42.9)	0
Decreased appetite	3 (23.1)	0	3 (60.0)	0	1 (33.3)	0	1 (14.3)	0
Dysgeusia	3 (23.1)	0	3 (60.0)	0	1 (33.3)	0	1 (14.3)	0
Headache	2 (15.4)	0	0	0	2 (66.7)	0	1 (14.3)	0
Dyspepsia	1 (7.7)	0	3 (60.0)	0	1 (33.3)	0	0	0
Thrombocytopenia	4 (30.8)	2 (15.4)	0	0	0	0	2 (28.6)	0
Hypokalemia	4 (30.8)	0	0	0	0	0	0	0
Upper respiratory tract infection	2 (15.4)	0	0	0	2 (66.7)	0	0	0
Abdominal distension	2 (15.4)	1 (7.7)	2 (40.0)	0	2 (66.7)	0	1 (14.3)	0
Muscle spasms	2 (15.4)	0	2 (40.0)	0	2 (66.7)	0	1 (14.3)	0
Vision blurred	1 (7.7)	0	2 (40.0)	0	2 (66.7)	0	0	0
Hypotension	4 (30.8)	2 (15.4)	1 (20.0)	0	0	0	0	0
Asthenia	2 (15.4)	0	1 (20.0)	0	1 (33.3)	0	2 (28.6)	0
Insomnia	2 (15.4)	0	3 (60.0)	0	0	0	0	0
Hypocalcemia	1 (7.7)	0	3 (60.0)	1 (20.0)	0	0	0	0
Tremor	1 (7.7)	0	3 (60.0)	0	0	0	0	0
Dysphonia	0	0	3 (60.0)	0	0	0	0	0
AST increased	1 (7.7)	0	1 (20.0)	0	0	0	2 (28.6)	1 (14.3)
Dyspnea exertional	1 (7.7)	0	1 (20.0)	0	0	0	2 (28.6)	1 (14.3)
Tachycardia	0	0	1 (20.0)	0	2 (66.7)	0	0	0
Edema	1 (7.7)	0	0	0	1 (33.3)	0	2 (28.6)	0
ALT increased	1 (7.7)	1 (7.7)	0	0	1 (33.3)	0	2 (28.6)	2 (28.6)
Hypertension	0	0	2 (40.0)	0	0	0	2 (28.6)	1 (14.3)
Fluid retention	0	0	0	0	0	0	2 (28.6)	0
Abdominal pain upper	1 (7.7)	0	0	0	0	0	3 (42.9)	0

The most common any-grade adverse events listed are those that occurred in ≥4 patients in the ORd, 5/14 cohort; or ≥3 patients in the ORd, 2/7 cohort; or ≥2 patients in the OCyd cohort; or ≥2 patients in the OMP cohort

*AE* adverse event, *ALT* alanine aminotransferase, *AST* aspartate aminotransferase, *OCyd* oprozomib, cyclophosphamide, and dexamethasone; *OMP* oprozomib, melphalan, and prednisone; *OPZ* oprozomib, *ORd* oprozomib, lenalidomide, and dexamethasone

In OPZ006, the mean duration of oprozomib exposure was 22.3 weeks (range, 1–53). TEAEs were reported for all OPZ006 patients (Table 1). Grade ≥ 3 TEAEs were reported in five patients (71.4%). Three patients (42.9%) had a TEAE that led to oprozomib discontinuation (supplement). Gastrointestinal AEs were among the most common TEAEs in OPZ003 and OPZ006. In the ORd 5/14 cohort, diarrhea, nausea, and vomiting rates were 84.6, 84.6, and 53.8%.

Among 21 treated OPZ003 patients (ORd 5/14, *n* = 13; ORd 2/7, *n* = 5; OCyd, *n* = 3), 15 achieved ≥PR (overall response rate [ORR], 71.4%) (Table 2); 38% achieved ≥very good PR. Two patients achieved a complete response (CR) and one a stringent CR. All achieved ≥stable disease. The time to response ranged from 22 to 98 days. ORR was 66.7% in ORd-treated patients (5/14, 61.5%; 2/7, 80.0%) and 100.0% in OCyd-treated patients. ORR was 100.0% for ORd- or OCyd-treated patients receiving ≥2 cycles. None of the patients experienced disease progression or death within the follow-up period; median (95% CI) follow-up times were 3.7 (0.7–4.4) months (ORd, 5/14 schedule), 11.4 (3.6–16.8) months (ORd, 2/7 schedule), and 11.2 (3.8–15.0) months (OCyd, 2/7 schedule).

**Table 2:** Treatment responses

	ORd		OCyd	OMP
	5/14 Schedule, 150–210 mg OPZ *N* = 13	2/7 Schedule, 210 or 240 mg OPZ *N* = 5	2/7 Schedule, 210 mg OPZ *N* = 3	2/7 Schedule, 180 mg OPZ *N* = 7
Best overall response, *n* (%)				
Stringent complete response	0	1 (20.0)	0	0
Complete response	2 (15.4)	0	0	1 (14.3)
Very good partial response	2 (15.4)	2 (40.0)	1 (33.3)	0
Partial response	4 (30.8)	1 (20.0)	2 (66.7)	2 (28.6)
Stable disease	1 (7.7)	0	0	3 (42.9)
Not evaluable	2 (15.4)	0	0	0
Missing or unknown	2 (15.4)	1 (20.0)	0	1 (14.3)
Overall response rate, %	61.5	80.0	100.0	42.9

*OCyd* oprozomib, cyclophosphamide, and dexamethasone; *OMP* oprozomib, melphalan, and prednisone; *OPZ* oprozomib; *ORd* oprozomib, lenalidomide, and dexamethasone

Among seven OMP-treated patients in OPZ006, ORR was 42.9% (Table 2). One patient achieved CR and two achieved PR.

In conclusion, although antimyeloma activity (including CRs) was demonstrated in NDMM patients, gastrointestinal toxicities and variable PK were concerns with the oprozomib formulation used in this study. Dose-escalation above the starting dose occurred on the 2/7 but not 5/14 schedule, possibly because this schedule was more tolerable. The 2/7 schedule is being taken forward in a phase 1b, dose-exploration study (NCT02939183) in RRMM that is evaluating the two new oprozomib formulations to determine whether a lower maximum plasma concentration could improve gastrointestinal tolerability and whether a faster dissolution could limit pharmacokinetic variability.

## Supplementary information

Supplemental Material
