# Supplementary material for: Oprozomib in patients with newly diagnosed multiple myeloma
Source: Blood Cancer J. 2019 Aug 16;9(9):66. doi: 10.1038/s41408-019-0232-6 (PMC6697695; doi:10.1038/s41408-019-0232-6)
Supplement: Supplementary file 1 — Supplemental Material [file 41408_2019_232_MOESM1_ESM.docx]

**Supplementary Methods**

*Concomitant medication*

In OPZ003, oral hydration (1.5 to 2 liters per 24 hours) was given to all patients 24 to 48 hours prior to initiation of therapy in every cycle, and this was continued throughout days of dosing. Premedication with allopurinol or other approved uric acid lowering agents and close monitoring was highly recommended for patients with high tumor burden (due to the risk of tumor lysis syndrome [TLS]). A proton-pump inhibitor (PPI; e.g., omeprazole or lansoprazole) was required (unless the patient had intolerance or hypersensitivity) for the duration of treatment to prevent peptic disease or other gastrointestinal toxicities. Antithrombotic agents were required for patients on the ORd arm, such as aspirin (or other anticoagulant or antiplatelet medication such as clopidogrel bisulfate, low-molecular-weight heparin, or warfarin). At least 24 hours prior to cycle 1 day 1 and for the duration of the study, the following medications were strongly recommended: Herpes zoster prophylaxis (with acyclovir, valacyclovir, or equivalent antiviral while taking oprozomib) and bisphosphonate therapy (such as pamidronate or zoledronic acid) for skeletal prophylaxis. Patients were to be premedicated with a 5-HT3 inhibitor, such as ondansetron or granisetron, at the first onset of nausea and/or vomiting prior to oprozomib dosing each day and throughout the day as needed to prevent nausea and vomiting. If nausea/vomiting at any grade persisted, aprepitant and additional antiemetics could be used if needed. For patients developing any grade of diarrhea, loperamide was strongly recommended at the onset of symptoms. For patients with persistent diarrhea despite the use of loperamide, the addition of diphenoxylate and atropine was strongly recommended. The use of concomitant medications was similar for patients in OPZ006.

*Data sharing*

Qualified researchers may request data from Amgen clinical studies. Complete details are available at the following: http://www.amgen.com/datasharing.

**Supplementary Results**

*Safety*

In OPZ003, AEs leading to treatment discontinuation in the ORd arm included pulmonary embolism, deep vein thrombosis, abdominal distension, abdominal pain, diarrhea, muscular weakness, syncope, nausea, gastrointestinal toxicity, and capillary leak syndrome (n=1 each). No deaths occurred in the OPZ003 study. In OPZ006, AEs that led to oprozomib discontinuation included cerebrovascular accident and pneumonia in one patient; diarrhea, dizziness, and vomiting in another patient; and pneumonia parainfluenzae viral in the third patient. No deaths occurred in the OPZ006 study.

**Figure S1** **Study design schemas for (A) OPZ003 study and (B) OPZ006 study**

**(A)**


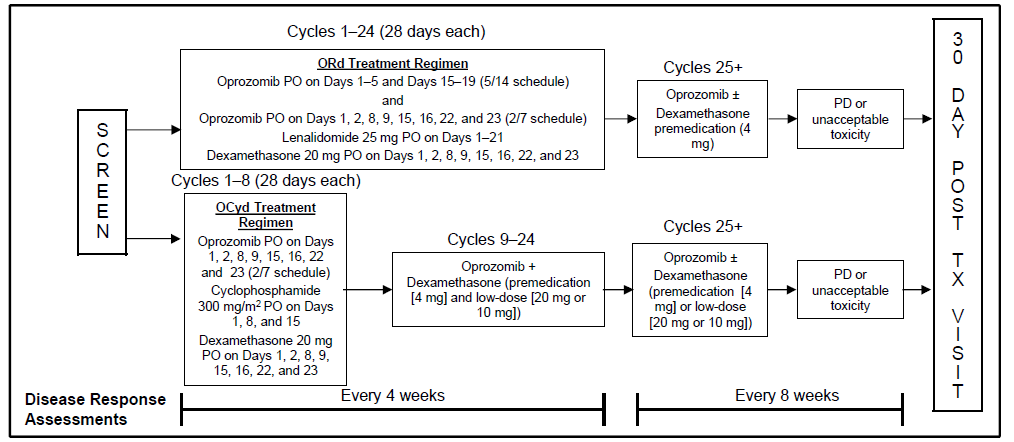


**(B)**


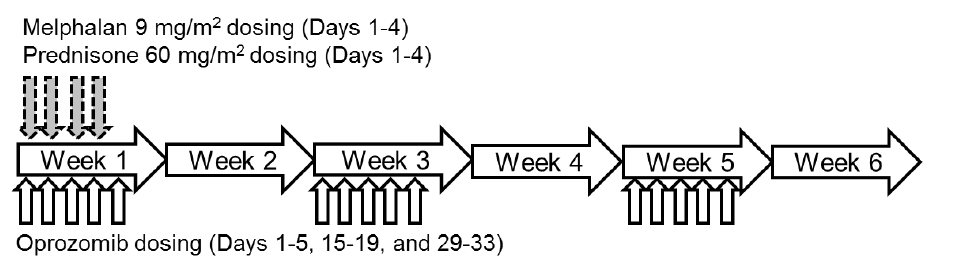


**Table S1 Patient baseline and disease characteristics by dose-level cohort**

| **Characteristic** | **OPZ003** | | | | | | | | | | | **OPZ006** |
| --- | --- | --- | --- | --- | --- | --- | --- | --- | --- | --- | --- | --- |
|  | **Cohort 5/14 Schedule**  **ORd** | | | | **Cohort 2/7 Schedule**  **ORd** | | | **Cohort 2/7 Schedule**  **OCyd** | | **Overall Total** | | **OMP** |
|  | **150 mg**  ***N*=3** | **180 mg**  ***N*=7** | **210 mg**  ***N*=3** | **Total**  ***N*=13** | **210 mg**  ***N*=3** | **240 mg**  ***N*=2** | **Total**  ***N*=5** | **210 mg**  ***N*=3** | **Total**  ***N*=3** | ***N*=21** | | **180 mg**  ***N*=7** |
| Median age, years (range) | 71.0  (67–79) | 71.0 (54–77) | 65.0 (62–67) | 67.0 (54–79) | 64.0 (54–76) | 60.0 (56–64) | 64.0 (54–76) | 69.0 (63–71) | 69.0 (63–71) | | 67.0 (54–79) | 71.0  (66–84) |
| Sex, *n* (%) |  |  |  |  |  |  |  |  |  | |  |  |
| Male | 3 (100.0) | 5 (71.4) | 1 (33.3) | 9 (69.2) | 3 (100.0) | 0 | 3 (60.0) | 3 (100.0) | 3 (100.0) | | 15 (71.4) | 3 (42.9) |
| Female | 0 | 2 (28.6) | 2 (66.7) | 4 (30.8) | 0 | 2 (100.0) | 2 (40.0) | 0 | 0 | | 6 (28.6) | 4 (57.1) |
| Race, *n* (%) |  |  |  |  |  |  |  |  |  | |  |  |
| American Indian/Alaska Native | 0 | 0 | 0 | 0 | 1 (33.3) | 0 | 1 (20.0) | 0 | 0 | | 1 (4.8) | 0 |
| Black | 0 | 1 (14.3) | 0 | 1 (7.7) | 0 | 0 | 0 | 0 | 0 | | 1 (4.8) | 0 |
| White | 3 (100.0) | 5 (71.4) | 3 (100.0) | 11 (84.6) | 2 (66.7) | 2 (100.0) | 4 (80.0) | 3 (100.0) | 3 (100.0) | | 18 (85.7) | 5 (71.4) |
| Other/Not reported | 0 | 1 (14.3) | 0 | 1 (7.7) | 0 | 0 | 0 | 0 | 0 | | 1 (4.8) | 2 (28.6) |
| ECOG PS, *n* (%) |  |  |  |  |  |  |  |  |  | |  |  |
| 0 | 1 (33.3) | 4 (57.1) | 3 (100.0) | 8 (61.5) | 1 (33.3) | 1 (50.0) | 2 (40.0) | 3 (100.0) | 3 (100.0) | | 13 (61.9) | 5 (71.4) |
| 1 | 2 (66.7) | 3 (42.9) | 0 | 5 (38.5) | 2 (66.7) | 1 (50.0) | 3 (60.0) | 0 | 0 | | 8 (38.1) | 1 (14.3) |
| 2 | 0 | 0 | 0 | 0 | 0 | 0 | 0 | 0 | 0 | | 0 | 1 (14.3) |

The baseline value is defined as the last available measurement taken before the first dose of any study drug.

ECOG PS, Eastern Cooperative Oncology Group performance status; OCyd, oprozomib in combination with cyclophosphamide and dexamethasone; ORd, oprozomib in combination with lenalidomide and dexamethasone.

**Table S2 Patient disposition**

|  | **OPZ003** | | | | | | | | | **OPZ006** |
| --- | --- | --- | --- | --- | --- | --- | --- | --- | --- | --- |
|  | **Cohort 5/14 Schedule**  **ORd** | | | | **Cohort 2/7 Schedule**  **ORd** | | | **Cohort 2/7 Schedule**  **OCyd** | **Overall Total** | **OMP** |
|  | **150 mg**  ***N*=3** | **180 mg**  ***N*=7** | **210 mg**  ***N*=3** | **Total**  ***N*=13** | **210 mg**  ***N*=3** | **240 mg**  ***N*=2** | **Total**  ***N*=5** | **210 mg**  ***N*=3** | ***N*=21** | **180 mg**  ***N*=7** |
| Number of patients enrolled^a^ | 3 (100.0) | 7 (100.0) | 3 (100.0) | 13 (100.0) | 3 (100.0) | 2 (100.0) | 5 (100.0) | 3 (100.0) | 22 (100.0) | 7 (100.0) |
| Number of patients who discontinued treatment,  *n* (%)^b^ | 2 (66.7) | 7 (100.0) | 3 (100.0) | 12 (92.3) | 2 (66.7) | 2 (100.0) | 4 (80.0) | 2 (66.7) | 18 (85.7) | 5 (71.4) |
| AE^c^ | 1 (33.3) | 1 (14.3) | 1 (33.3) | 3 (23.1) | 0 | 1 (50.0) | 1 (20.0) | 0 | 4 (19.0) | 3 (42.9) |
| Physician decision | 0 | 0 | 0 | 0 | 1 (33.3) | 1 (50.0) | 2 (40.0) | 1 (33.3) | 3 (14.3) | 1 (14.3) |
| Progression of disease | 0 | 0 | 0 | 0 | 0 | 0 | 0 | 0 | 0 | 0 |
| Withdrawal by patients^d^ | 0 | 3 (42.9) | 1 (33.3) | 4 (30.8) | 0 | 0 | 0 | 1 (33.3) | 5 (23.8) | 0 |
| Other^d^ | 1 (33.3) | 3 (42.9) | 1 (33.3) | 5 (38.5) | 1 (33.3) | 0 | 1 (20.0) | 0 | 6 (28.6) | 1 (14.3) |

^a^Percentage relative to the number of patients enrolled.

^b^Percentage relative to the number of patients treated/dosed.

^c^AEs leading to discontinuation in the ORd treatment arm included pulmonary embolism, deep vein thrombosis, abdominal distension, abdominal pain, diarrhea, muscular weakness, syncope, nausea, GI toxicity, and capillary leak syndrome (n=1 patient each). For OMP, AEs leading to oprozomib discontinuation included cerebrovascular accident and pneumonia in one patient; diarrhea, dizziness, and vomiting in one patient; and pneumonia parainfluenzae viral.

^d^ORd 5/14 schedule: Of the nine patients who discontinued due to “other” reasons or “withdrawal by patient”, five went off study treatment due to forthcoming stem cell transplant, three withdrew due to low grade toxicities (primarily nausea and diarrhea), and one was withdrawn due to a greater than 4-week interruption of study treatment; ORd 2/7 schedule: The one patient discontinued due to “other” reasons which were related to non-treatment related back pain due to a prior fall and nausea while on oprozomib alone; OCyd 2/7 schedule: The one patient who withdrew did so because of depressed mood and low-grade diarrhea.

AE, adverse event; GI, gastrointestinal; OCyd, oprozomib in combination with cyclophosphamide and dexamethasone; OMP, oprozomib in combination with melphalan and prednisone; ORd, oprozomib in combination with lenalidomide and dexamethasone.

**Table S3** **Dose-limiting toxicities by preferred term (safety population)^a^**

|  | **ORd** | | | | **ORd** | | | **OCyd** | |
| --- | --- | --- | --- | --- | --- | --- | --- | --- | --- |
|  | **5/14 Schedule** | | | | **2/7 Schedule** | | | **2/7 Schedule** | |
|  | **150 mg OPZ**  ***N*=3** | **180 mg OPZ**  ***N*=7** | **210 mg OPZ^b^**  ***N*=3** | **Total**  ***N*=13** | **210 mg OPZ**  ***N*=3** | **240 mg OPZ**  ***N*=2** | **Total**  ***N*=5** | **210 mg OPZ**  ***N*=3** | **Total**  ***N*=3** |
| Number of patients reporting dose-limiting toxicities, *n* (%) | 2 (66.7) | 2 (28.6) | 2 (66.7) | 6 (46.2) | 0 | 1 (50.0) | 1 (20.0) | 0 | 0 |
| Capillary leak syndrome | 0 | 0 | 0 | 0 | 0 | 1 (50.0)^c^ | 1 (20.0) | 0 | 0 |
| Syncope | 0 | 0 | 2 (66.7)^d^ | 2 (15.4) | 0 | 0 | 0 | 0 | 0 |
| Abdominal distension | 0 | 1 (14.3) | 0 | 1 (7.7) | 0 | 0 | 0 | 0 | 0 |
| Abdominal pain | 0 | 1 (14.3) | 0 | 1 (7.7) | 0 | 0 | 0 | 0 | 0 |
| Alanine aminotransferase increased | 1 (33.3) | 0 | 0 | 1 (7.7) | 0 | 0 | 0 | 0 | 0 |
| Hypophosphatemia | 1 (33.3) | 0 | 0 | 1 (7.7) | 0 | 0 | 0 | 0 | 0 |
| Hypotension | 0 | 1 (14.3) | 0 | 1 (7.7) | 0 | 0 | 0 | 0 | 0 |
| ^a^Dose-limiting toxicities were defined as occurring during the 28-day period of cycle 1 combination therapy.  ^b^The 210 mg dose was the starting dose in the 5/14 schedule. The dose was de-escalated 2 times to 180 and 150 mg.  ^c^Manifested as fever, hypotension, hypoxia, liver function test derangement, unresponsiveness, and pneumonia.  ^d^Diarrhea and dehydration was coincident with both syncope events and bradycardia in one case.  OCyd, oprozomib in combination with cyclophosphamide and dexamethasone; ORd, oprozomib in combination with lenalidomide and dexamethasone. | | | | | | | | | |
